# Why Is Colorectal Cancer Occurring Earlier? Metabolic Dysfunction, Underrecognized Carcinogens, and Emerging Controversies

**DOI:** 10.1007/s13679-026-00700-z

**Published:** 2026-03-20

**Authors:** Maria Dalamaga, Sofia Rozani, Dimitra Petropoulou

**Affiliations:** 1 Department of Biologic Chemistry, Medical School, National and Kapodistrian University of Athens, Athens, Greece; 2 2nd Department of Surgery, Aretaieio University Hospital, National and Kapodistrian University of Athens, Athens, Greece

**Keywords:** Colorectal Cancer, Epigenetic acceleration, Early-onset colorectal cancer, Exposome, Gut microbiome, Human papillomavirus, Insulin resistance, Metabolic dysfunction, Obesity, Risk Factor, Western lifestyle

## Abstract

**Purpose of the review:**

Early-onset colorectal cancer (EOCRC), defined as colorectal cancer diagnosed before 50 years of age, is increasing worldwide and represents a major challenge to current prevention and screening paradigms. While obesity and metabolic dysfunction are important contributors, EOCRC appears to arise from a complex interplay of genetic susceptibility, metabolic stress, early-life exposures, microbiome alterations, environmental factors, and potentially infectious agents. This review aims to critically examine recent epidemiologic, molecular, and multi-omics evidence on EOCRC etiology, integrate metabolic and exposomic hypotheses within a life-course framework, highlight ongoing controversies and methodological limitations, and discuss emerging challenges for risk stratification and prevention.

**Recent Findings:**

Recent EOCRC-specific meta-analyses and cohort studies demonstrate moderate associations between EOCRC and obesity, central adiposity, insulin resistance, metabolic syndrome, and lifestyle factors such as alcohol use, sedentary behavior, and ultra-processed diets. Molecular and multi-omics studies reveal that EOCRC is enriched for chromosomal instability–driven tumors, metabolic and inflammatory signaling pathways, gut microbiome dysbiosis, and accelerated epigenetic aging, exceeding chronological age by more than a decade in some patients. In utero exposures, early-life anthropometry and metabolic dysfunction may contribute to EOCRC risk, potentially through developmental programming of metabolic and inflammatory pathways. Complementing metabolic dysfunction, growing attention has focused on the modern exposome, including antibiotics, micro- and nanoplastics, smoking, environmental pollutants, ionizing radiation exposure, and chronic infections. Notably, HPV has emerged as a potential, though still controversial, cofactor in EOCRC, with heterogeneous tissue-detection studies and limited age-stratified epidemiologic data underscoring the need for further investigation. Clinically, EOCRC remains largely symptom-driven, with frequent diagnostic delays and symptom misattribution contributing to advanced-stage presentation.

**Summary:**

EOCRC is a multifactorial malignancy driven by cumulative metabolic dysfunction and inflammatory stress interacting with environmental, microbial, and early-life exposures on a background of genetic susceptibility and accelerated epigenetic aging. Addressing the EOCRC epidemic will require integrated, life-course prevention strategies that move beyond age-based screening to incorporate metabolic health optimization, lifestyle and exposome modification, improved symptom recognition, and risk-adapted early detection.

**Supplementary Information:**

The online version contains supplementary material available at 10.1007/s13679-026-00700-z.

## Introduction

Colorectal cancer (CRC) remains one of the most prevalent and lethal malignancies worldwide, ranking as the third most frequently diagnosed cancer and the second leading cause of cancer-related mortality, with more than 1.9 million new cases and over 900,000 deaths annually [1, 2]. Historically, CRC has been considered a disease of older adults; however, over the past several decades, a consistent epidemiologic shift has emerged. The incidence of CRC diagnosed before the age of 50, early-onset colorectal cancer (EOCRC), has increased steadily across multiple high-income countries, while CRC incidence in older adults has stabilized or declined [3, 4]. EOCRC now accounts for a substantial and growing proportion of CRC cases (10–13%) and deaths (7%) in several countries, underscoring its public health relevance [5]. Globally, CRC represented the most frequent early-onset gastrointestinal malignancy reported in 2022 (54.3%), followed by gastric (23.8%), esophageal (13.2%) and pancreatic cancers [6].

Temporal analyses have revealed a strong birth-cohort effect. Between the late 1980s and mid-2010s, the age-adjusted incidence of EOCRC increased by approximately 60–65%, with continued annual increases of more than 2% in recent years [7, 8]. Individuals born around 1990 experience substantially higher age-specific EOCRC risk compared with earlier cohorts, suggesting that generational changes in environmental, metabolic, and behavioral exposures, rather than aging or screening practices alone, are driving this trend [9]. According to the most recent U.S. cancer statistics, CRC incidence in individuals younger than 50 years is increasing by approximately 2.9% per year, with a steeper rise exceeding 6% per year after 2019, while CRC mortality, despite continuing to decline overall, has begun to increase by about 1% annually among adults younger than 55 years [10]. Similar increases are now being reported in regions undergoing rapid Westernization, including parts of East Asia, indicating that EOCRC is a global phenomenon rather than one confined to Western populations [3, 11]. In parallel, EOCRC prevalence has increased substantially, rising by an estimated 56% between 1990 and 2019, from 16.3 to 25.4 cases per 100,000 population [12].

Clinically, EOCRC differs from late-onset CRC (LOCRC) in several aspects. Younger patients are more likely to be diagnosed following symptom onset rather than screening, often experience prolonged diagnostic delays, and frequently present with advanced-stage disease. Tumors are disproportionately located in the distal colon and rectum, and display partially distinct molecular features compared with late-onset disease [13, 14]. Although hereditary cancer syndromes and family history confer substantial individual risk, pathogenic germline variants (PGV) account for a minority of EOCRC cases and cannot explain the rapid population-level increase in incidence [15]. These observations highlight fundamental limitations of traditional, age-based and genetically centered risk models.

Accumulating evidence supports a multifactorial, life-course model of EOCRC etiology. Strong birth-cohort effects, combined with the long latency required for colorectal carcinogenesis, point toward early-life biological vulnerability that precedes clinical disease by decades. Metabolic dysfunction, including obesity, insulin resistance, and type 2 diabetes mellitus (T2DM), has risen sharply among younger generations and is consistently associated with increased EOCRC risk [16]. Metabolic dysfunction interacts with diet and physical inactivity, gut microbiome dysbiosis, environmental and chemical exposures, early-life medical interventions (such as antibiotics), and possibly infectious agents, converging on chronic inflammatory and metabolic stress, and accelerated epigenetic aging [17]. As a result, current prevention and screening strategies, largely extrapolated from LOCRC, may be insufficient for addressing EOCRC risk.

Although studies on EOCRC have expanded rapidly, most existing reviews address individual risk domains in isolation, such as genetics, obesity, microbiome alterations, or screening, often extrapolating from late-onset disease. Few reviews have integrated genetic susceptibility with epigenetic acceleration, metabolic dysfunction, gut microbiome dysbiosis, environmental and infectious exposures, and life-course timing within a unified etiologic and translational model. This review addresses this gap by synthesizing EOCRC-specific epidemiologic, clinical, molecular, and multi-omics evidence to propose an integrated life-course framework of EOCRC etiology, with implications for risk stratification, prevention, and early detection. Within this framework, metabolic dysfunction is positioned as a central and well-supported biological substrate, while environmental, infectious, and exposomic factors are considered complementary and potentially synergistic modifiers whose relative contribution may vary across populations and life stages. We further highlight methodological limitations, unresolved controversies, and priority areas for future EOCRC research. To the best of our knowledge, this is among the first reviews to explicitly integrate metabolic dysfunction, exposomic exposures, epigenetic acceleration, and multi-omics signatures into a unified life-course model specific to EOCRC rather than extrapolating from late-onset disease.

## Literature Search

For this narrative review, a literature search was conducted using PubMed/MEDLINE, Scopus, and Web of Science up to January 2026. Search terms included combinations of Medical Subject Headings (MeSH) and keywords such as “early-onset colorectal cancer,” “colorectal cancer” “obesity,” “metabolic syndrome,” “insulin resistance,” “microbiome,” “microplastics,” “persistent organic pollutants,” “human papillomavirus,” “radiation,” “epigenetic age acceleration,” “diet,” “antibiotics”, “exposome”, “virus”, etc. Priority was given to EOCRC-specific meta-analyses, prospective cohort studies, multicenter genomic and multi-omics investigations, and large population-based analyses. Reference lists of relevant reviews and key publications were manually screened to identify additional pertinent studies. Given the rapidly evolving nature of EOCRC research, priority was given to studies published from 2018 onward, while earlier seminal works were included when relevant. Finally, we acknowledge that all literature cannot be covered in the context of this review.

## Limitations of Traditional Risk Factors in Early-Onset Colorectal Cancer

Traditional risk factors for CRC are well established and include male sex, Caucasian race, increasing age, the presence of adenomatous polyps, hereditary cancer syndromes, family history and inflammatory bowel disease (IBD) [18, 19]. Most CRCs are sporadic, with approximately 20% showing familial clustering and only about 5% attributable to well-defined hereditary syndromes such as Lynch syndrome or familial adenomatous polyposis (FAP) [15]. Accordingly, family history has long been considered a central determinant of CRC risk and a key driver of intensified screening strategies in younger individuals.

However, recent high-quality evidence indicates that the magnitude of CRC risk conferred by family history may be lower than previously assumed, particularly when derived from cohort-based data. In a large systematic review and meta-analysis including 42 case-control and 20 cohort studies, individuals with one first-degree relative (FDR) with CRC had a pooled relative risk (RR) of 1.92 in case-control studies but only 1.37 in cohort studies. For individuals with two or more affected FDRs, the corresponding RRs were 2.81 and 2.40, respectively. The highest risk was observed among individuals whose FDR was diagnosed with CRC before the age of 50 years, with pooled RRs of 3.57 in case-control studies and 3.26 in cohort studies. These findings underscore the modifying role of study design, number of affected relatives, and age at diagnosis in interpreting familial risk [15].

Importantly, EOCRC-specific meta-analytic data show a substantially stronger association between family history and EOCRC, with a pooled OR of 5.90 (95% CI 3.67–9.48), underscoring the particular relevance of familial aggregation in early-onset disease [18]. Similarly, FDRs with cancer have a 70% increased risk of developing EOCRC based on a large-scale cohort study from China [20]. Nonetheless, despite this elevated risk, family history still accounts for a minority of EOCRC cases at the population level. Similarly, associations between EOCRC and the personal history of other malignancies, such as breast cancer, identify select high-risk subgroups but do not account for the broader epidemiologic trends [21].

In the context of EOCRC, additional distinctions further challenge traditional risk models. Tumors in younger patients more frequently arise in the distal colon and rectum, with rectal involvement in up to 41% of men and 36% of women, and are often diagnosed at more advanced stages compared with later-onset CRC [19]. These patterns reflect both biological differences and prolonged diagnostic delay rather than screening failure alone. Molecular profiling has revealed a predominance of microsatellite-stable tumors and global DNA hypomethylation, reflected by hypomethylation of LINE-1, suggesting biological features that differ from those typically observed in older patients [22]. EOCRC tumors instead appear enriched for alternative molecular trajectories, including *TP53* and *PTEN* alterations and adverse histological features, such as poor differentiation, mucinous phenotype, and signet-ring histology [23]. These characteristics indicate that EOCRC may follow distinct carcinogenic pathways that are not fully captured by classical genetic or adenoma-centric models.

IBD, encompassing ulcerative colitis and Crohn’s disease, remains a strong and well-recognized risk factor for CRC and is particularly relevant in younger populations [14]. EOCRC-specific pooled analyses indicate that IBD confers an increased risk of EOCRC of approximately 4.4 (pooled OR 4.43, 95% CI 4.05–4.84) [18]. However, despite high individual-level risk, IBD accounts for a relatively small fraction of EOCRC cases at the population level, consistent with registry-based estimates [24]. Thus, IBD represents a high-risk clinical subgroup rather than a primary driver of the EOCRC epidemic.

Demographic factors also modify EOCRC risk [18]. EOCRC-specific meta-analyses indicate that male sex is associated with a modestly increased risk (pooled OR ~ 1.20), suggesting a role as a risk modifier rather than a primary etiologic factor. Higher EOCRC incidence observed among individuals of Caucasian ethnicity likely reflects differences in exposure profiles, healthcare access, and diagnostic practices, rather than intrinsic biological susceptibility, and should therefore be interpreted with caution.

Overall, these observations underscore the limitations of traditional CRC risk factors in explaining the current EOCRC epidemic. While hereditary syndromes, family history and IBD remain essential components of individual risk assessment and clinical management, they fail to account for the majority of EOCRC cases and the pronounced birth-cohort effects observed in recent epidemiologic studies. These limitations provide a strong rationale for expanding etiologic models beyond classical genetic and clinical risk factors to include metabolic, environmental and early-life exposures, which are increasingly implicated in EOCRC.

## The Metabolic Trap: Obesity, Insulin Resistance, Diet and Gut Dysbiosis

### Obesity, Insulin Resistance and EOCRC

A growing body of evidence implicates metabolic dysfunction as a central contributor to CRC and EOCRC. Notably, temporal trends in EOCRC incidence have closely paralleled the expanding burden of obesity and T2DM amid younger adults since the 1970s [25, 26]. A meta-analysis including 242,561 CRC cases, of which 32,275 were diagnosed at ≤ 55 years of age, showed that overweight and obesity confer ORs of 1.32 (95% CI 1.19–1.47) and 1.88 (95% CI 1.40–2.54) respectively, for EOCRC, with childhood and adolescent obesity further increasing risk.

 [27]. Prospective cohort studies similarly showed that overweight, obesity, and weight gain since early adulthood are more strongly associated with CRC diagnosed before age 50 than with later-onset disease [28, 29].

Recent Mendelian randomization and pooled analyses indicate that increased body size, assessed by body mass index (BMI), waist circumference, and body fat percentage, is causally associated with elevated risk for most molecular subtypes of CRC, including chromosomal instability, microsatellite instability, CpG island methylator phenotype–defined tumors and common mutations (*KRAS*,* BRAF*), with the exception of Lynch syndrome–associated tumors, where no association is observed [30, 31]. Notably, these studies also show that the magnitude of the association is stronger for the serrated and alternate pathways compared to the traditional adenoma–carcinoma pathway [31]. While obesity is a risk factor for most sporadic CRC subtypes, its contribution alone is insufficient to explain EOCRC incidence patterns [21].

Central adiposity and the cumulative number of metabolic syndrome components demonstrate dose–response relationships with EOCRC, particularly for distal colon and rectal cancers [32]. A recent EOCRC-specific systematic review and meta-analysis further reinforces the relevance of metabolic dysfunction, reporting significant associations for obesity (OR ~ 1.5), overweight (OR ~ 1.2), abdominal obesity (OR ~ 1.2), elevated triglycerides (OR ~ 1.1), hypertension (OR ~ 1.2), and metabolic syndrome (OR ~ 1.3) [18]. Lifestyle factors closely linked to metabolic dysfunction, including alcohol consumption, smoking, sedentary behavior, Western dietary patterns, and sugar-sweetened beverage (SSBs) intake, were also consistently associated with increased EOCRC risk. Collectively, these findings support an additive model in which multiple modest metabolic and lifestyle exposures accumulate across the life course to accelerate colorectal carcinogenesis [18].

Diabetes mellitus has also been associated with EOCRC risk. A recent meta-analysis including 33,359 EOCRC cases and 14,259,289 controls reported a significant association between diabetes and EOCRC (OR 1.43; 95% CI 1.08–1.8), with stronger effects observed among individuals with unmanaged disease, while no significant association was seen in those with well-controlled diabetes [33]. Large prospective cohort studies corroborate these findings, demonstrating increased EOCRC risk among individuals diagnosed with diabetes before age 50 [34, 35]. The association is independent of other metabolic risk factors being consistent across diverse populations. In the UK Biobank cohort, increased glycemia (> 7.0 mmol/L), a characteristic of T2DM, was associated with a 61% elevated risk of EOCRC and a 14% increased risk of LOCRC [36]. Notably, the effect size for T2DM and EOCRC is modest compared with the association observed for obesity and EOCRC, but is similar to the effect size for LOCRC. These modest effect sizes likely reflect heterogeneity in disease duration, glycemic control, and treatment, as well as underdiagnosis of diabetes in younger populations.

Overall, diabetogenic processes characterized by insulin resistance and hyperinsulinemia are associated with increased odds of CRC, including EOCRC [37]. Biologically, obesity promotes a pro-tumorigenic milieu characterized by chronic low-grade inflammation, insulin resistance, hyperinsulinemia and elevated insulin-like growth factor-1 (IGF-1), which promote carcinogenesis via altered gene expression, colonic epithelial proliferation, anti-apoptotic signaling and pro-inflammatory cytokine and adipocytokine production [38]. In particular, metabolic derangements associated with excess adiposity activate proliferative and anti-apoptotic signaling via the PI3K–AKT–mTOR and RAS–MAPK pathways. Concurrent hyperglycemia induces oxidative stress, DNA damage, and aberrant gene regulation. Obesity and T2DM further exacerbate systemic and local inflammation through altered adipokine secretion, macrophage polarization, and impaired intestinal barrier function [39, 40].

Finally, emerging evidence suggests that metabolic vulnerability may be programmed early in life. Higher maternal BMI (≥ 30 kg/m²) and higher birth weight (≥ 4000 g) have been associated with increased CRC risk in the offspring (adjusted hazard ratio/aHR ~ 2.5 and RR per 1000 g increase of ~ 1.1 respectively), with a substantial proportion of cases occurring before age 50 [41, 42]. These findings suggest that in utero exposures, early-life anthropometry and metabolic dysfunction may contribute to EOCRC risk, potentially through developmental programming of metabolic and inflammatory pathways [41].

### Dietary Patterns, Specific Food Components, Alcohol Intake and EOCRC

Beyond adiposity, dietary patterns independently modulate EOCRC risk. Although most meta-analyses are not EOCRC-specific, birth-cohort effects and prospective data suggest that long-term exposure to adverse dietary patterns characterized by a high intake of refined carbohydrates, saturated fats and low fiber beginning in adolescence or early adulthood may be particularly relevant for EOCRC development [43–45]. In the Nurses’ Health Study II, women in the highest quintile of Western dietary pattern had a significantly increased risk of early-onset high-risk colorectal adenomas (OR 1.67, 95% CI 1.18–2.37), which are established precursors of EOCRC [45]. This pattern was characterized by high intake of processed and red meats, butter, high-fat dairy products, eggs and refined grains.

In particular, poor diet quality promotes chronic inflammation, insulin resistance, and gut microbiome dysbiosis, all of which are implicated in the pathogenesis of EOCRC [4]. Western and high-fat diets increase systemic markers of hyperinsulinemia and chronic inflammation, including C-peptide, C-reactive protein (CRP), interleukin (IL)-6, and tumor necrosis factor-α (TNF-α), creating a metabolic milieu conducive to tumorigenesis [46]. Notably, experimental studies in murine models suggest that genetic susceptibility may interact with diet, as illustrated by loss-of-function variants in transcriptional regulators that predispose to polyp formation only under high-fat dietary conditions, mediated by reduced Cdx2 expression and enhanced β-catenin signaling [47].

Beyond overall dietary patterns, individual food components may contribute to colorectal carcinogenesis either directly or indirectly through their effects on obesity and T2DM. Ultra-processed foods (UPFs), characterized by extensive industrial processing and the inclusion of manufactured ingredients and additives, now account for up to 60% of total daily energy intake in many high-income countries. An increased intake of UPFs is associated with obesity, T2DM, MASLD and obesity-related cancer [48–51]. Recent cohort analyses indicate that high UPF consumption is associated with an increased risk of conventional adenomas, serrated lesions, and high-risk polyps, even after adjustment for BMI and overall diet quality, supporting an independent contribution to colorectal carcinogenesis [52]. Based on 3 large US cohorts, high consumption of UPFs is associated with a 29% increased risk of CRC in men, especially for distal colon cancer, independent of BMI and overall diet quality [53]. Among 29,105 women under 50 years of age participating in the Nurses’ Health Study II, high UPF consumption was associated with a ~ 45% higher odds of early-onset conventional adenomas, whereas no significant association was observed for serrated lesions [54].

Processed meat and SSBs have been consistently associated with increased EOCRC risk. Meta-analytic data indicate that processed meat intake confers an OR of ~ 1.5, while SSB consumption is associated with ORs of ~ 1.5–2.0, with stronger associations observed for distal and rectal tumors [18, 55, 56]. These associations appear to be independent of total caloric intake and adiposity, underscoring the role of dietary composition beyond energy balance alone.

Finally, alcohol intake is a well-established risk factor for EOCRC. Meta-analyses have shown that high alcohol consumption increases EOCRC risk by 39–56%, with a dose-response relationship observed for both frequency and quantity [57–59]. These associations are consistent across sex and tumor subsites, with stronger effects for distal colon and rectal cancers [58]. Alcohol consumption may promote CRC through several interrelated mechanisms, including acetaldehyde-driven genotoxicity, particularly in individuals with reduced aldehyde dehydrogenase activity [60], reactive oxygen and nitrogen species–mediated tissue damage, alterations in folate metabolism, and alcohol-induced gut microbiome dysbiosis [58].

Collectively, these findings indicate that dietary patterns and specific food components contribute to EOCRC risk independently of obesity, acting through sustained metabolic stress, chronic inflammation, and gut dysbiosis. Importantly, dietary exposures often begin early in life and persist for decades, providing a plausible explanation for the strong birth-cohort effects observed in EOCRC incidence.

### Gut microbiome Dysbiosis, Antibiotic Exposure and EOCRC

The gut microbiota and its metabolites play a central role in colorectal carcinogenesis and are increasingly implicated in EOCRC. EOCRC is characterized by distinct patterns of gut dysbiosis, including enrichment of pro-inflammatory and potentially genotoxic bacterial taxa such as *Fusobacterium nucleatum*, polyketide synthase–positive (pks⁺) *Escherichia coli*, which encodes colibactin, a genotoxin that induces DNA double-strand breaks, *Parvimonas micra*, and *Flavonifractor plautii* [61]. These microbial alterations are thought to mediate dietary and lifestyle effects by promoting mucosal inflammation, epithelial injury, and DNA damage, with mediation analyses linking specific taxa to processed meat, fried food and snack consumption. Importantly, several of these taxa have been linked to distal and rectal tumors, consistent with the anatomic distribution of EOCRC [61].

Beyond taxonomic shifts, multi-omics studies integrating metagenomics and metabolomics have identified EOCRC-specific functional microbial signatures, distinct from those observed in later-onset CRC. These include alterations in microbial metabolic pathways related to bile acid, tryptophan, and choline metabolism, as well as enrichment of microbial genes associated with red meat consumption and inflammatory signaling [62]. These findings suggest that early-life and long-term exposures may shape a distinct microbial ecosystem that contributes to premature colorectal carcinogenesis.

**Fig. 1:**
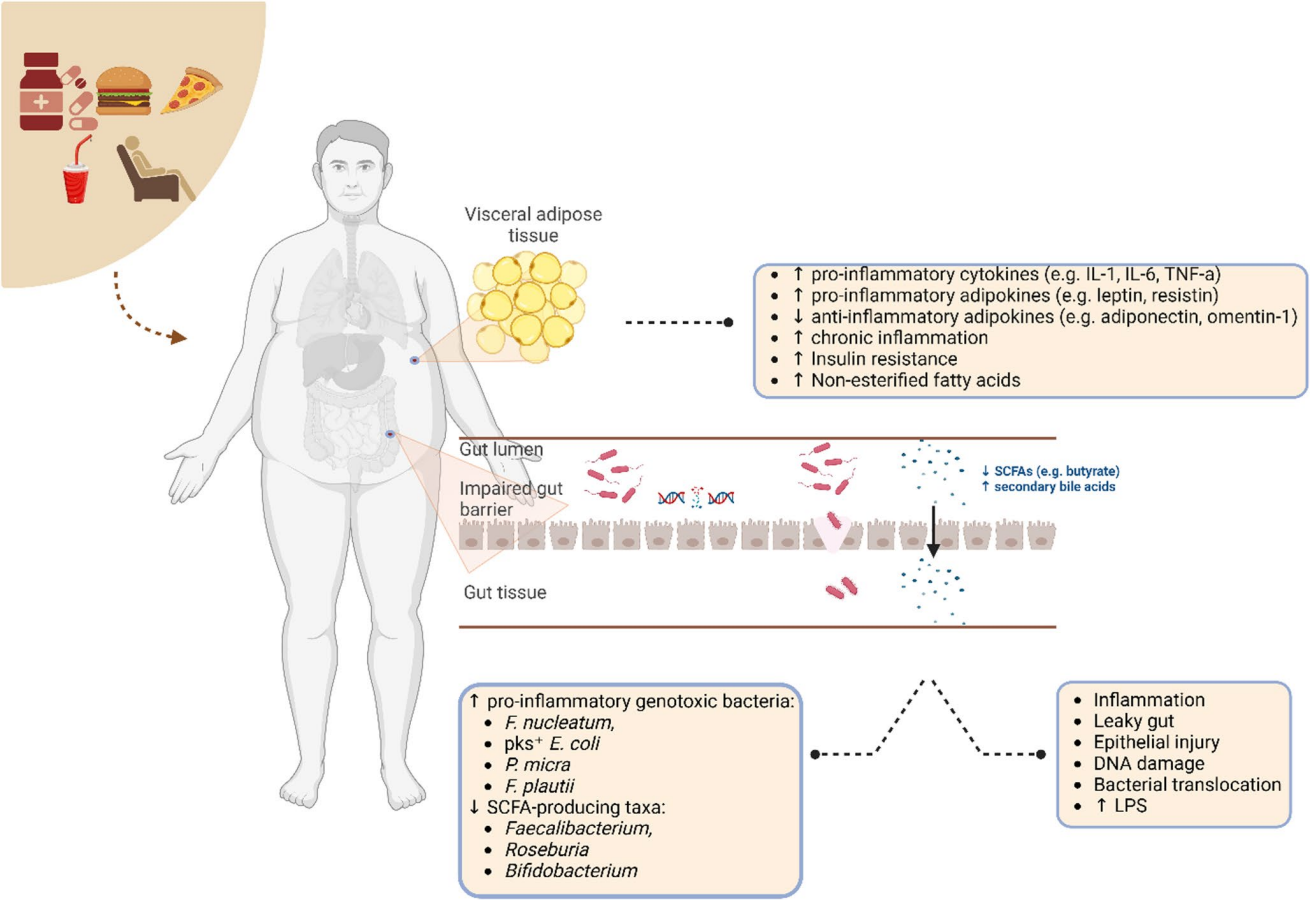
**legend.** Metabolic dysfunction and gut dysbiosis may promote early-onset colorectal cancer through interconnected metabolic, inflammatory, and microbial mechanisms. Abbreviations: IL: interleukin, LPS: lipopolysaccharide; SCFA: Short-chain fatty acids; TNF-α: Tumor Necrosis Factor-alpha. Created in Biorender by Dimitra Petropoulou (January 27, 2026) https://BioRender.com/zjs3yy2

Microbial metabolites represent key functional mediators linking dysbiosis to EOCRC. Short-chain fatty acids (SCFAs), particularly butyrate, exert anti-inflammatory and barrier-protective effects in the colon, whereas dysbiosis associated with obesity and Western dietary patterns is characterized by the depletion of SCFA-producing taxa (such as *Faecalibacterium*,* Roseburia*, and *Bifidobacterium*) and the enrichment of opportunistic and oral-derived pathogens, and pro-inflammatory metabolites [63]. Notably, *Fusobacterium nucleatum* has been shown to activate β-catenin signaling, upregulate pro-inflammatory cytokines (e.g. TNF and IL-17), and suppress antitumor immune responses through binding to inhibitory receptors on immune cells [64–66]. Alterations in bile acid metabolism further link metabolic dysfunction and gut microbiota to EOCRC risk, as increased exposure to secondary bile acids, such as lithocholic acid, has been associated with epithelial injury and chronic inflammation [67]. Together, these metabolic shifts may lower the threshold for malignant transformation in the susceptible colonic epithelium (Fig. 1).

Antibiotic exposure represents a clinically relevant and potentially modifiable driver of gut dysbiosis that may contribute to obesity etiopathogenesis and EOCRC susceptibility by inducing long-lasting alterations in microbial diversity, bile acid metabolism and pro-inflammatory signaling [68, 69]. Large observational studies and meta-analyses have shown modest but consistent associations between cumulative antibiotic use and increased CRC risk, with pooled ORs generally ranging from ~ 1.1 to 1.2 for the highest exposure categories [70–72]. The association is strongest for broad-spectrum, anti-anaerobic and penicillin-class antibiotics, and is more pronounced for colon cancer than rectal cancer [70, 73]. EOCRC-specific studies further suggest that long-term or recurrent antibiotic exposure, particularly during early life, is associated with increased risk of EOCRC (adjusted OR 1.49, 95% CI 1.07–2.07) and early-onset adenomas [73, 74]. The association appears to be dose-dependent, with longer duration and higher cumulative prescriptions conferring greater risk. However, some studies report no significant association between antibiotic use in adulthood and EOCRC, suggesting that timing of exposure may be critical. The heterogeneity across studies likely reflects differences in exposure timing, antibiotic class, indication, and cumulative dose, underscoring the importance of early-life windows of susceptibility. Importantly, antibiotic exposure may also interact with diet and metabolic status, amplifying dysbiosis and inflammatory signaling over time.

Overall, gut microbiome dysbiosis and antibiotic exposure form a self-reinforcing pathway linking diet, metabolic dysfunction, and early-life medical interventions to EOCRC risk. Rather than acting as isolated risk factors, these exposures appear to modify host–microbe interactions across the life course, contributing to epithelial barrier disruption, chronic inflammatory stress, immune dysregulation and genotoxic stress leading to increased colorectal vulnerability decades before cancer diagnosis.

## The Modern Exposome: Smoking and Hidden Environmental and Chemical Drivers

Although effect sizes for individual environmental exposures are generally smaller and more heterogeneous than those observed for obesity-related metabolic dysfunction, the early-life onset, ubiquity, and cumulative nature of environmental and chemical drivers warrant particular consideration within a life-course model.

Cigarette smoking is a well-established risk factor for CRC and has also been implicated in EOCRC, particularly when exposure begins in adolescence or early adulthood. A recent meta-analysis reported that current smokers had a significantly increased risk of EOCRC compared with never-smokers (OR 1.33, 95% CI 1.17–1.52), whereas former smokers did not exhibit a statistically significant excess risk [75]. These findings are supported by additional meta-analyses and pooled cohort studies demonstrating a dose–response relationship with smoking intensity and duration, while mechanistic evidence implicates tobacco-associated carcinogens in DNA damage, epigenetic alterations, chronic inflammation, and microbiome perturbations that may promote colorectal tumorigenesis at younger ages [18, 29, 76, 77].

With regard to air pollution, direct epidemiologic evidence linking exposure to EOCRC remains limited. Nevertheless, available evidence suggests that air pollution, particularly fine particulate matter (PM2.5), NO₂, and SO₂, is associated with increased CRC risk and mortality in the general population, with risk estimates generally ranging from 1.1 to 2.3 for the highest exposure categories after adjustment for confounders such as smoking and socioeconomic status [78, 79]. Although age-stratified EOCRC estimates are scarce, the early-life onset and chronic nature of air pollution exposure support biological plausibility for a contributory role in EOCRC [78], particularly in urbanized and rapidly industrializing regions.

Micro- and nanoplastics (MNPs) are widespread environmental contaminants detected across food, water, and biological matrices, with human exposure occurring through ingestion, inhalation, and dermal contact with the potential for systemic distribution and tissue accumulation [12]. MNPs are increasingly recognized as potential contributors to EOCRC through several mechanistic pathways. Human biomonitoring studies have confirmed systemic exposure, with plastic polymers detected in blood and gastrointestinal tissues, while experimental studies demonstrate that oral exposure to MNPs may disrupt the intestinal barrier integrity, impair the colonic mucus layer, and induce chronic low-grade inflammation [80, 81]. In animal models, MNPs exacerbate colitis-associated colorectal tumorigenesis and promote pro-tumorigenic immune alterations, including increased pro-inflammatory macrophage activity and T-cell exhaustion within the colonic microenvironment [82, 83]. Mechanistic studies further have shown that MNPs may induce oxidative stress, DNA damage and gut microbiota dysbiosis, and may act as vectors for co-exposure to other environmental carcinogens, thereby amplifying cytotoxic and carcinogenic effects [81]. Human biomonitoring studies have identified higher concentrations of MNPs in colorectal tumor tissues compared with adjacent non-neoplastic mucosa. Case–control studies further report a dose–response association between fecal microplastic burden and CRC risk (adjusted OR 11.3; 95% CI 6.77–19.5, p for trend < 0.01), with a particular association in females and those who frequently eat high-fat or spicy foods [84]. However, these estimates derive from cross-sectional or case–control designs with potential for reverse causation, measurement heterogeneity, and residual confounding, and therefore should not be interpreted as indicating a stronger causal contribution than established metabolic risk factors. While EOCRC-specific evidence is still emerging, the early-life onset, persistence, and bioaccumulative nature of MNP exposure raise concern for long-term colorectal vulnerability.

Persistent organic pollutants (POPs), including organochlorine pesticides and related compounds, have also been linked to colorectal neoplasia [85]. These chemicals are characterized by environmental persistence, bioaccumulation due to their lipophilic nature, endocrine-disrupting properties, toxicity and carcinogenic potential [86]. Large ecological studies in the USA have reported associations between PFAS in drinking water and increased incidence of digestive system cancers overall, but not specifically for EOCRC; however, these findings are limited by study design and lack of individual-level exposure data [87]. Prospective biomonitoring studies have reported increased CRC risk associated with higher circulating concentrations of specific POPs, with hazard ratios increasing across exposure tertiles [88]. Despite supportive mechanistic data [89], evidence linking per- and polyfluoroalkyl substances (PFAS) to CRC remains mixed, with recent meta-analyses reporting no statistically significant association overall with no observed dose-response relationship [90]. Moreover, animal studies have shown that PFAS exposure may increase intestinal tumorigenesis, but results are inconsistent across models and compounds [91]. Taken together, current evidence suggests that certain POPs may act as cumulative risk modifiers rather than dominant carcinogenic drivers.

Pesticide exposure has been associated with increased CRC risk in occupational cohorts and meta-analyses, particularly for specific herbicides and insecticides, such as chlorpyrifos with rectal cancer and aldicarb with colon cancer [92]. Reported RRs for colon and rectal cancer generally range from ~ 1.2 to 1.3, depending on compound class and exposure intensity, with some studies specifically noting an elevated risk in younger populations and in regions with high pesticide use [93–95]. Although EOCRC-focused analyses are limited, early-life and cumulative exposures to pesticides remain biologically plausible contributors within a life-course framework, especially in agricultural and high-use settings.

Finally, evidence linking medications other than antibiotics to EOCRC remains limited. Aspirin and non-steroidal anti-inflammatory drugs (NSAIDs) are consistently associated with a 20–30% reduction in overall CRC risk [96, 97], while a recent EOCRC-focused meta-analysis found no significant association, with substantial heterogeneity [18]. Proton pump inhibitors show no consistent association with CRC in meta-analyses, with any signal mainly confined to prolonged use (> 5 years) and residual confounding concerns [98]. Metformin has been linked to reduced adenoma formation and CRC incidence in diabetic populations, but EOCRC-specific data are lacking [99]. Statins, hormone replacement therapy, oral contraceptives, and antihypertensives have not shown significant associations with EOCRC, with evidence limited by small study numbers and lack of age-stratified analyses [97]. Overall, chemopreventive evidence is strongest for aspirin and NSAIDs, while data for other drug classes remain inconclusive for EOCRC.

Collectively, modern environmental and chemical exposures represent a diffuse but potentially important component of EOCRC risk. Individual effect sizes are modest, but the ubiquity, early-life onset, cumulative nature of these exposures may result in meaningful population-level impact, particularly when acting in concert with metabolic dysfunction and gut dysbiosis. These considerations support the inclusion of the exposome in EOCRC etiologic and preventive models.

## Emerging and Underrecognized Carcinogenic Exposures

### Ionizing Radiation

Direct epidemiologic evidence linking ionizing radiation specifically to EOCRC remains limited; however, substantial human data demonstrate an association between radiation exposure and increased CRC risk across multiple exposure contexts. Long-term follow-up of atomic bomb survivors has shown a clear dose–response relationship for colon cancer incidence, with an excess RR per Gy of 0.63 (95% CI 0.34–0.98), persisting decades after exposure. Importantly, these associations were observed independently of age at exposure, underscoring the long latency of radiation-related colorectal carcinogenesis [100]. Following the Chernobyl nuclear accident, population-based studies in Finland and Sweden reported a modest but statistically significant association between fallout-related radiation exposure and increased colon cancer incidence among women, while no consistent effect was observed for rectal cancer or in men [101, 102].

Evidence from medically exposed populations further supports the relevance of radiation to EOCRC. Childhood and young-adult cancer survivors who received abdominopelvic radiotherapy have a substantially increased risk of subsequent CRC, with reported ORs of ~ 3.0 among higher-dose groups and median latencies of more than two decades [103, 104]. Similarly, survivors of Hodgkin lymphoma treated with subdiaphragmatic radiotherapy demonstrate a significantly increased CRC risk, with a clear dose–response relationship and synergistic effects observed with procarbazine [104]. These cohorts provide a biologically and temporally plausible link between early-life radiation exposure and EOCRC. By contrast, evidence linking low-dose diagnostic radiation exposure (including CT and X-ray imaging) to EOCRC is indirect. Meta-analyses in adult populations demonstrate increased overall cancer risk with higher cumulative diagnostic radiation exposure [105], but CRC- and EOCRC-specific outcomes have not been consistently evaluated. While prenatal and low-dose diagnostic exposures remain difficult to quantify, repeated imaging beginning at young ages may contribute to cumulative mutational burden. Overall, radiation exposure appears most relevant to EOCRC when exposure occurs early in life and involves abdominal or pelvic fields.

### Infectious Viral Agents

Human papillomavirus (HPV), a well-established oncogenic virus in anogenital malignancies, has been repeatedly detected in colorectal tissues, raising the possibility that it may also contribute to a subset of EOCRC via persistent mucosal infection, chronic inflammation, and E6/E7-mediated interference with p53/RB signaling [106]. Multiple meta-analyses and large observational studies have reported higher HPV DNA detection rates, especially high-risk genotypes such as HPV16 and HPV18, in CRC tissue compared with controls, with pooled ORs ranging from ~ 2 to > 10, depending on geographic region and detection methodology [107–109]. Importantly, tissue-detection–based ORs reflect presence of viral DNA rather than prospective infection exposure, and may overestimate etiologic contribution compared with longitudinal epidemiologic risk estimates. The association is strongest in Asian and South American populations and in studies using PCR-based detection methods [107, 108]. Despite these observations, EOCRC-specific, age-stratified epidemiologic data remain limited, and p16INK4a expression, a marker of transcriptionally active HPV, is inconsistently detected in HPV-positive CRC samples [110]. Population-based cohort data suggest that individuals with documented HPV infection may have an increased risk of CRC, particularly for rectal and rectosigmoid tumors, varying by anatomical site, sex, and comorbidities, including diabetes, hypertension and abnormal liver function [111, 112]. Taken together, current evidence supports biological plausibility but does not establish causality, highlighting the need for prospective EOCRC-focused studies incorporating tumor genotyping, exposure history, and vaccination status.

Beyond HPV, other viruses have been detected in CRC tissues, including JC polyomavirus (JCV), Epstein–Barr virus (EBV), cytomegalovirus (HCMV), and torque teno virus (TTV). Meta-analyses have reported elevated odds of viral detection in CRC tissue compared with normal mucosa, ranging from 3.4 for EBV, 6.59 for the presence of HCMV to 11 for JCV T-antigen; however, most studies rely on tissue-based detection rather than prospective infection history and lack age-stratified EOCRC analyses [113–115]. Finally, recent virome studies have identified enrichment of TTV, an anellovirus, in CRC tissues, suggesting a possible oncogenic role, though the clinical significance and mechanistic pathways remain to be clarified [116]. Overall, these findings remain hypothesis-generating and insufficient to support a causal role in EOCRC.

Current evidence linking SARS-CoV-2 infection or vaccination to EOCRC incidence is insufficient. Available studies primarily examine cancer outcomes among patients with pre-existing malignancy or short-term temporal associations without establishing causality [117, 118]. At present, available data remain insufficient to implicate SARS-CoV-2 infection or mRNA vaccination in EOCRC etiology, beyond indirect pathways such as inflammation, immune dysregulation, microbiome dysbiosis, and the well-documented pandemic-related delays in screening and diagnosis [119].

### *Helicobacter pylori* and Chronic Infection

*Helicobacter pylori* infection has been consistently associated with colorectal neoplasia overall, with meta-analyses reporting pooled ORs of ~ 1.4–1.8 for CRC and colorectal adenomas [120, 121]. Although EOCRC-specific analyses are limited, some studies suggest higher prevalence of *H. pylori* infection among younger CRC patients and stronger associations for left-sided tumors [122]. Interestingly, eradication therapy appears to reduce CRC risk (OR 0.44), but causality and mechanisms remain under investigation [123].

Taken together, emerging and underrecognized carcinogenic exposures, including radiation and chronic infections, highlight the importance of early-life and cumulative biological stress in EOCRC pathogenesis. While current evidence does not support a dominant role for any single exposure, their interaction with metabolic dysfunction, immune dysregulation, and long latency carcinogenesis reinforces the need for life-course–oriented EOCRC research.

## Genetic Susceptibility Meets Epigenetic Acceleration

Epigenetic age acceleration may represent a mechanistic bridge linking inherited susceptibility with cumulative metabolic, environmental, and inflammatory exposures across the life course. Genetic susceptibility represents an important component of EOCRC risk. PGVs, most commonly involving mismatch repair genes associated with Lynch syndrome, polyposis syndromes, and DNA repair pathways, are identified in up to ~ 20–25% of EOCRC cases [19]. Twin and familial aggregation studies further support a substantial inherited contribution, with heritability estimates exceeding 30% and monozygotic twin concordance rates of 15–20% [124]. Genome-wide association studies have additionally identified EOCRC-associated risk loci dominated by noncoding variants enriched in regulatory regions active in gastrointestinal tissues, supporting a polygenic architecture in which gene–environment interactions play a central role [124].

Despite this genetic contribution, inherited susceptibility alone cannot account for the rapid rise in EOCRC incidence observed across birth cohorts. The magnitude and temporality of the EOCRC epidemic are incompatible with purely genetic explanations, particularly given the long latency required for colorectal carcinogenesis. Instead, these observations point toward early and persistent environmental and metabolic perturbations that interact with genetic background to accelerate disease onset.

Epigenetic acceleration, defined as a mismatch between biological (epigenetic) age and chronological age, has emerged as a defining feature of EOCRC. DNA methylation–based analyses demonstrate that biological age in EOCRC patients exceeds chronological age by approximately 10–15 years, providing quantitative evidence of accelerated aging in early-onset disease [125, 126]. Importantly, epigenetic age acceleration is detectable not only in tumor tissue but also in adjacent normal colonic mucosa, indicating that epigenetic alterations precede malignant transformation and interact with inherited susceptibility rather than simply reflecting tumor biology [125, 126]. Blood-based epigenetic clocks similarly demonstrate positive associations between epigenetic age acceleration and incident CRC risk, with OR of 1.20–1.44 per decile increase, in population-based studies [127].

Colorectal-specific methylomic studies further show that age-related methylation drift occurs earlier and progresses more rapidly in EOCRC, suggesting accelerated proliferative history and prolonged premalignant evolution [128]. Modeling of these patterns indicates that founder premalignant clones may arise early in life and persist for decades before progression to invasive cancer, particularly in distal and rectal tumors [128].

In particular, early-life exposures are increasingly recognized as key drivers of epigenetic reprogramming. Prenatal and perinatal factors, including maternal obesity, metabolic status, mode of delivery, infant feeding practices (e.g. plastic bottles), and early antibiotic exposure, may induce durable epigenetic and immune alterations that increase colorectal vulnerability later in life. These early imprints may be reinforced during childhood and adolescence by Western dietary patterns, excess adiposity, sedentary behavior, and repeated inflammatory insults. Together, these observations support a model in which epigenetic acceleration serves as an integrative readout of cumulative genetic and environmental risk.

Twin studies further reinforce the importance of non-genetic influences, as monozygotic twins discordant for cancer often exhibit substantial differences in epigenetic age despite shared genetic background [129]. In addition, mosaic epigenetic inheritance, including constitutional epimutations affecting mismatch repair genes, may underlie EOCRC in the absence of detectable coding mutations, further blurring the distinction between genetic and environmental causation [130].

In summary, genetic susceptibility and epigenetic acceleration provide a unifying model for EOCRC pathogenesis, reconciling modest individual effect sizes of environmental and metabolic exposures with the substantial population-level increase in EOCRC incidence. This model highlights epigenetic aging as a central mediator linking early-life exposures, cumulative biological stress, and premature colorectal carcinogenesis. Thus, epigenetic acceleration can be conceptualized not as an isolated phenomenon, but as an integrative biological readout of the cumulative exposome acting on genetically susceptible individuals.

## Societal and Behavioral Determinants of Early-Onset Colorectal Cancer

Sedentary lifestyle, low physical activity, sleep disruption, circadian rhythm dysregulation, and chronic psychosocial stressors have emerged as potential contributors to EOCRC, although the strength of EOCRC-specific evidence varies across these factors. Among behavioral factors, sedentary behavior and physical inactivity have the most consistent EOCRC-specific support. In the Nurses’ Health Study II, prolonged television viewing, a surrogate marker of sedentary lifestyle, was associated with a significantly increased risk of EOCRC, independent of BMI and leisure-time physical activity, with particularly strong associations for rectal cancer [131]. A systematic review and meta-analysis similarly reported an increased EOCRC risk associated with sedentary behavior (OR ~ 1.2) [18]. Conversely, higher levels of physical activity were associated with a reduced risk of EOCRC in prospective European cohort data (HR ~ 0.7) [29]. Importantly, these associations persist after adjustment for diet and adiposity, supporting an independent contribution of sedentary behavior to EOCRC risk. Given the early adoption and persistence of sedentary habits across the life course, these behaviors may contribute to cumulative colorectal vulnerability.

Evidence linking sleep disruption and circadian rhythm dysregulation to EOCRC is more limited and largely indirect. Meta-analyses evaluating colorectal neoplasia overall report associations between abnormal sleep duration, obstructive sleep apnea, and night-shift work, and increased CRC risk; however, most studies do not provide EOCRC-specific, age-stratified estimates [132, 133]. Nevertheless, disruption of circadian regulation may plausibly contribute to EOCRC through effects on metabolic regulation, immune function, and inflammatory signaling, particularly when exposure begins early in adulthood. In addition, experimental and epidemiologic evidence suggests that light-at-night–induced suppression of melatonin, a hormone with oncostatic and immunomodulatory effects in the colorectum, may provide a biologically plausible mechanistic link [131, 132].

Data on chronic psychological and social stressors are similarly sparse for EOCRC specifically. Large prospective cohorts and meta-analyses have reported modest associations between perceived stress, work-related stress, and CRC incidence or mortality, with pooled RRs of ~ 1.3–1.4, particularly in North American populations [134, 135]. These associations are substantially confounded by health behaviors and socioeconomic factors, making causal inference challenging. Although direct prospective data for EOCRC remain sparse, chronic psychological and social stressors may activate the hypothalamic–pituitary–adrenal axis, leading to sustained cortisol exposure, neuroendocrine dysregulation and low-grade systemic inflammation [136–138]. Chronic stress may act as a risk modifier by promoting metabolic dysfunction, systemic inflammation, and adverse health behaviors over time.

Importantly, behavioral and psychosocial factors intersect with structural determinants of health that influence early detection and outcomes. Despite improvements in CRC screening uptake overall, substantial socioeconomic and racial disparities persist. Racial and ethnic minority populations as well as uninsured or underinsured individuals are significantly less likely to undergo timely diagnostic evaluation, and are more likely to be diagnosed with advanced-stage EOCRC, resulting in poorer outcomes [139, 140]. These disparities reflect systemic barriers, including limited access to primary care, lower health literacy, and delayed referral for colonoscopy. In the context of EOCRC, where diagnosis is often symptom-driven rather than screening-based, such barriers may substantially amplify disease burden.

**Fig. 2:**
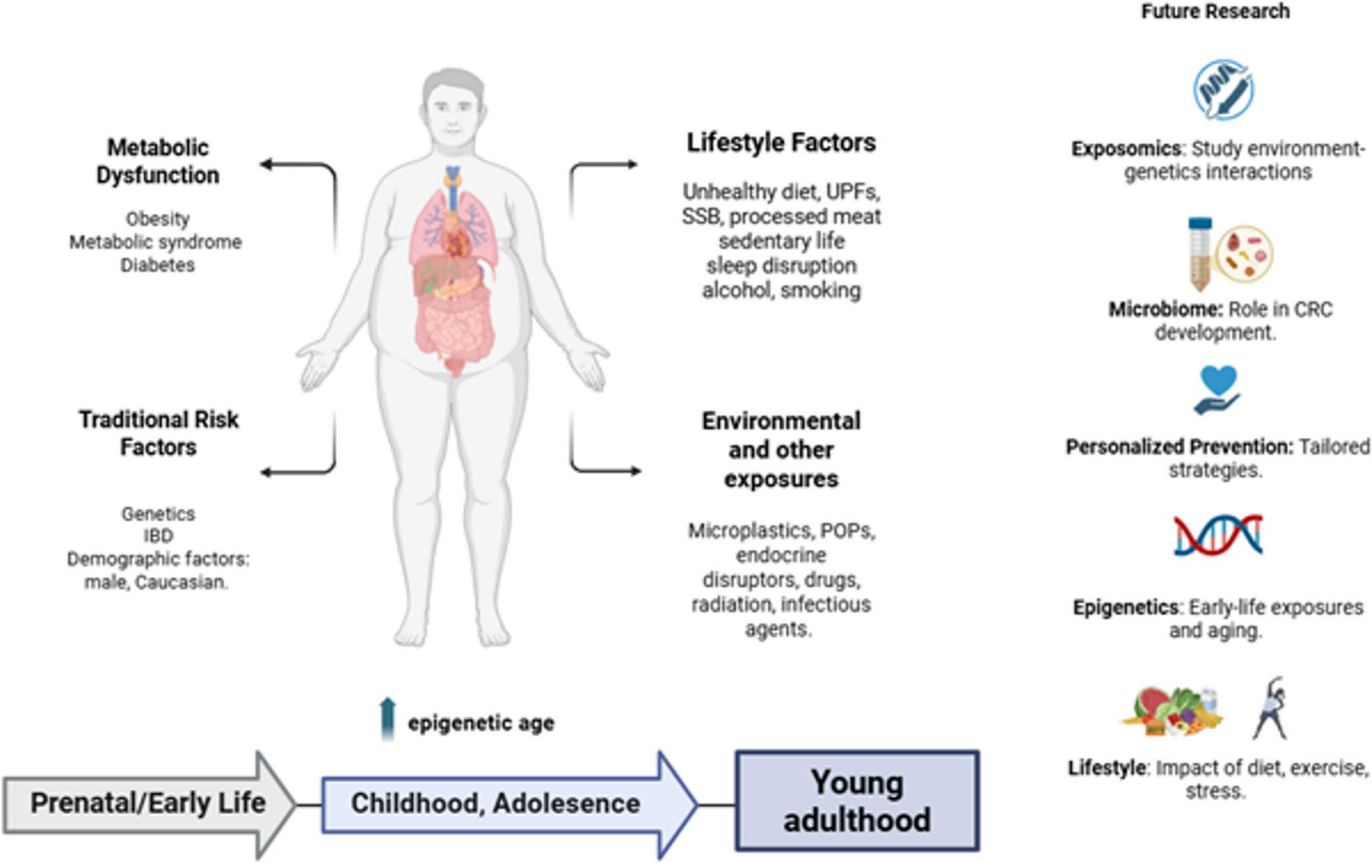
Multifactorial model of early-onset colorectal cancer showing interactions between traditional risk factors, environmental exposures, metabolic dysfunction, gut dysbiosis, epigenetic acceleration and lifestyle behaviors driving CRC risk in younger adults

Overall, societal and behavioral determinants influence EOCRC risk through both biological and healthcare-access pathways. While effect sizes for individual factors are modest, their early-life onset, persistence, and interaction with metabolic and environmental exposures reinforce the need for prevention strategies that extend beyond individual behavior change to address structural inequities and diagnostic delay. Figure 2 illustrates a life-course, multifactorial model of EOCRC, integrating genetic susceptibility with metabolic dysfunction, environmental exposures, gut dysbiosis, epigenetic acceleration, and societal and behavioral determinants.

Table 1 summarizes the major risk factors implicated in EOCRC, the strength of evidence supporting each association, and representative effect sizes derived from EOCRC-specific or EOCRC-relevant studies. Importantly, risk factors classified as “strong” or “moderate” are supported by EOCRC-specific meta-analyses or large prospective cohorts, whereas those categorized as “limited” or “emerging” should be interpreted as hypothesis-generating and requiring further validation. Moreover, the table highlights that no single exposure shows a dominant effect, but rather that EOCRC risk arises from the additive and potentially synergistic accumulation of multiple low-to-moderate effect factors across the life course. Genetic susceptibility, obesity and metabolic dysfunction, alcohol consumption, sedentary behavior, smoking, and gut microbiome dysbiosis emerge as the most consistently supported contributors, while environmental exposures, early-life programming factors, antibiotics, radiation, and chronic infections appear to act as risk modifiers whose population-level impact may be substantial despite modest individual effect sizes. This synthesis reinforces the conceptual model of EOCRC as a multifactorial disease driven by cumulative biological stress rather than a single dominant cause. 

**Table 1:** Risk factors for EOCRC and strength of evidence

Risk factor	EOCRC evidence-based?	Strength of evidence	Best human evidence types	Representative effect sizes (OR/RR/HR)	Comments	Representative studies
Demographic factors	Yes	Moderate	Meta-analysis	-OR 1.20 (male vs. female) -OR 1.44 (Caucasian)	- May reflect differences in body fat distribution, hormonal milieu, lifestyle exposures (alcohol, smoking) -May reflect geographic study representation, lifestyle exposures, screening access and healthcare utilization rather than ethnicity per se.	[18]
Genetic susceptibility (PGVs: Lynch/polyposis/DNA repair)	Yes	Strong	Multicenter germline sequencing (EOCRC)	PGVs in up to ~ 25% of EOCRC	Explains a minority of EOCRC; most EOCRC remains non-hereditary	[19]
Family history (1st-degree relative) of CRC Family history (1st -degree relative) of cancer	Yes Yes	Strong Moderate	Meta-analysis Large-scale prospective study	OR 4.21–5.90 HR 1.70	-High clinical relevance for screening initiation/intensity, but does not explain most EOCRC cases.	[18, 141] [20]
IBD	Yes	Strong (EOCRC age-stratified)	Meta-analysis -Registry cohorts; IBD surveillance cohorts; umbrella reviews	-OR 4.43 -Markedly ↑ CRC risk in long-standing/extensive colitis	A major established risk factor; EOCRC age-stratified reporting is inconsistent.	[18, 142]
Obesity (BMI)	Yes	Moderate-Strong	Meta-analysis + prospective cohorts	-OR 1.52 (obesity) -OR 1.18 (overweight)	Consistent; stronger when adiposity begins early. Mechanistically linked to insulin/IGF-1, chronic low-grade inflammation, adipokines.	[18]
Insulin resistance / metabolic dysfunction (incl. metabolic syndrome, central adiposity)	Yes	Moderate	Meta-analysis + Prospective cohorts; pooled cohort analyses; MR supportive	-OR 1.22 (abdominal obesity) -OR 1.29 (Mets) -Dose–response with MetS components and WC -OR 1.16 (HTN) -OR 1.12 (hypertriglyceridemia)	Supports insulin/IGF–chronic inflammation axis, adipokine dysregulation	[18, 32]
Diabetes	Yes	Moderate	Meta-analysis + prospective cohort studies	HR 1.43	Supports insulin/IGF–inflammation axis, adipokine dysregulation	[33]
Maternal BMI and birth weight	Indirect EOCRC-relevant	Emerging–Moderate	Prospective birth cohort; offspring cancer follow-up; meta-analyses (CRC overall)	- HR ~ 2.5 Maternal obesity associated with increased offspring CRC risk - RR 1.09 per 1000 g of birthweight. Higher birth weight associated with increased CRC risk	EOCRC-specific stratification limited, but ~ 50% of offspring CRC cases occurred < 50 years; supports early-life metabolic programming hypothesis.	[41, 42]
Dietary patterns (Western/low fiber etc.)	Yes	Moderate	Meta-analysis, Prospective cohorts; diet pattern analyses; adenoma surrogate endpoints	-OR 1.43 for Western dietary patterns -OR 1.67 Western pattern ↑ risk of EO high-risk adenomas, precursors of EOCRC -OR 0.64 DASH ↓ risk of EO high-risk adenomas, precursors of EOCRC -OR 0.55 AMED ↓ risk of EO high-risk adenomas, precursors of EOCRC -OR 0.71 AHEI ↓ risk of EO high-risk adenomas, precursors of EOCRC -Pattern-dependent; EOCRC endpoints often underpowered; early-life diet often missing	-Poor diet quality was associated with an increased risk of early-onset distal and rectal adenomas of high malignant potential as well as EOCRC - strong plausibility via metabolic/microbial pathways.	[18, 45]
Food groups	Yes	Moderate	Meta-analysis	-OR 1.53 for processed meat intake -OR 1.10 for red meat intake -OR 1.55 for sugar-sweetened beverages		[18]
Alcohol	Yes	Moderate-Strong	EOCRC-focused meta-analyses + prospective cohorts	-OR 1.39 (alcohol use, dose-response association) -OR 1.02 per 10 g/d ethanol increase - HR ~ 1.15 per 30 g/day in pooled cohorts for EOCRC	Mechanisms: acetaldehyde genotoxicity, ROS/RNS, folate dysregulation, dysbiosis	[29, 59]
Gut microbiome dysbiosis & metabolites	Partly	Moderate	Human metagenomics/metabolomics; mediation analyses; tumor/adjacent tissue; biomarker classifier studies	-EOCRC taxa/function signatures distinct from LOCRC -enriched *Flavonifractor plauti* and increased tryptophan, bile acid and choline metabolism in EOCRC	Strong coherence; causality still challenged by reverse causation/confounding	[61, 62]
Antibiotic exposure	Yes (EOCRC studies exist)	Moderate (inconsistent across settings)	Large population-based case-control; EHR cohorts	-OR 1.49 for early-onset CRC cohort -OR 1.09 for ≥ 50 years CRC -antibiotics not associated with rectal cancer -Effect size varies by class/window; microbiome modifier	Heterogeneity by antibiotic class, timing, indication	[73]
Smoking	Yes	Moderate	Meta-analysis + pooled cohorts	-OR 1.39–1.44 current; dose/intensity effect - HR 1.24 for smoking in pooled cohorts for EOCRC	Former smoking often weaker/non-significant depending on analysis.	[18, 29, 59, 75]
Air pollution (PM2.5/NO₂/SO₂)	Indirect for EOCRC	Limited–Emerging	Meta-analysis; Population cohorts; CRC incidence/mortality	RR 1.42 for CRC per 10 µg/m3 increment in PM2.5 exposure	EOCRC-specific analyses remain sparse	[78, 79]
Microplastics/nanoplastics (MNPs)	Early human + experimental, indirect for EOCRC	Emerging	Animal models + human tissue/fecal case-control	-OR 11.3 for CRC in the highest exposure quartile of MPs -Association particularly pronounced amid females and subjects who consumed spicy or high-fat foods -Higher tumor tissue/fecal MPs in CRC; dose–response in case-control	Promising but early; measurement heterogeneity and temporality limitations.	[81–84]
Persistent organic pollutants (POPs)	Mostly indirect for EOCRC	Limited–Emerging	-Meta-analysis, biomonitoring cohorts (CRC overall); community-based prospective cohort; mechanistic toxicology	-RR 0.83 (95% CI, 0.65–1.06) for PFOA and CRC - RR 0.71 (95% CI, 0.22–2.27) for PFOS and CRC - HR 2.68–7.43 for CRC (for the highest exposure tertiles for specific compounds of POPs) in a prospective Korean cohort	-No association between PFAS/PFOS exposure and CRC risk -exposome plausibility - CRC associations depend on compound class; EOCRC rarely examined	[88, 90]
Pesticides	Mostly indirect	Limited–Emerging	Meta-analysis; Occupational cohorts; weight-of-evidence reviews; biomonitoring; ecological studies	- For herbicides: RR 1.20–1.29 for colon cancer -For insecticides: RR 1.32 for colon cancer and RR 1.21 for rectal cancer - Risk estimates based on lifetime-days and intensity-weighted exposure.	- CRC risk signals for some pesticide classes -EOCRC-specific evidence limited -Heterogeneity (exposure assessment, confounding, mixtures).	[93]
Radiation (A-bomb, Chernobyl/fallout, iatrogenic RT, diagnostic CT/X-ray)	Indirect for EOCRC (strongest for early-life iatrogenic RT)	Moderate (CRC overall) / Strong (survivors with abdominopelvic RT)	Life Span Study cohort; fallout registries; survivor late-effects; dose–response meta-analyses	-excess RR per Gy 0.63 for colon cancer in atomic bomb survivors -OR 3.1 for a subsequent CRC in childhood cancer survivors after ART -Rate ratio 2.4 for CRC in survivors of HL following subdiaphragmatic RT; median interval between HL and CRC was 25.7 years	-EOCRC framing: strongest relevance is young-exposed cohorts (childhood RT, early-life exposures) where latency can still yield EOCRC. -dose-response relationship	[100, 103, 104]
HPV	EOCRC-specific evidence limited	Limited–Emerging (heterogeneous)	Systematic reviews/meta-analyses of HPV detection in CRC tissue; selected cohorts	-OR 2.39–10.78 for CRC and RR up to 2.97 based on HPV tissue-detection in CRC tissues vs. control tissues -Strongest associations in Asian and South American population and in PCR-based detection methods	-Methodological heterogeneity and need for stratified EOCRC data (vaccination, tumor HPV, anal/rectal infection). -Meta-analyses report HPV detection association with CRC, but results vary by geography, methods, and contamination control	[107–109, 143]
Other viruses (non-HPV; incl. SARS-CoV-2)	Indirect	Limited	Tissue-detection meta-analyses; Mostly associative/biologic plausibility; COVID–CRC outcome meta-analysis	-For JCV, pooled OR 10.95 for CRC -For EBV, pooled OR 3.4 for CRC -For CMV, pooled OR 6.59	Hypothesis-generating; signals exist but causality/EOCRC specificity unproven; SARS-CoV-2 evidence mainly outcomes/biology, not EOCRC incidence.	[113–115, 117]
*Helicobacter pylori* infection	Indirect	Limited–Emerging	Systematic reviews and meta-analyses of CRC/adenomas; case–control studies	-OR 1.49 for overall colorectal neoplasia (CRC or adenoma) -OR 1.59 for CRC - OR 1.47 for colorectal adenoma --OR 1.77 for advanced colorectal adenoma - OR 0.44 anti-*H. pylori* treatment (eradication)	-EOCRC-specific data lacking; biologically plausible via chronic inflammation, metabolic dysregulation, microbiome and epigenetic effects. - anti-*H. pylori* treatment on CRC incidence and mortality need large-scale, RCTs with prolonged follow-up	[123]
Sedentary behavior (screen time/TV)	Yes	Moderate	Prospective cohort + EOCRC meta-analysis	-For TV viewing: RR 1.69 for > 14 h/week vs. low (EO CRC) -For sedentary life: OR 1.24	One of the clearest EOCRC behavioral signals even after BMI/activity adjustment	[18, 131]
Physical activity	Yes	Moderate	Pooled prospective cohorts	HR 0.71 for colon cancer; protective	-Often independent of diet/adiposity. -Effects appear stronger for colon vs. rectal in pooled analyses.	[29]
Sleep disruption / circadian dysregulation	Indirect	Limited–Emerging	CRC overall meta-analyses (sleep duration, shift work); large-scale prospective cohort; EOCRC rarely stratified	-For long vs. moderate sleep duration, OR 1.33 for colorectal neoplasms -For sleep apnea, OR 1.75 for colorectal neoplasms - For ≥ 15 years night shifts, HR 1.20 for CRC - For ≥ 15 years night shifts, HR 2.69 for IRS2-positive tumors	EOCRC age-stratified outcomes usually absent; plausible pathway with limited EOCRC-specific quantification.	[132, 133]
Chronic psychosocial stressors	Indirect	Limited	Large cohorts/meta-analyses for CRC incidence/mortality; confounding	-Work stress: RR 1.36 for overall CRC -A statistically significant effect of work stress on CRC in North America (RR 1.51), but not significant in Europe	Risk modifier (via HPA axis, behaviors, metabolic risk, access to care), not a stand-alone causal driver	[134, 135, 138]

*AHEI* Alternate Healthy Eating Index, *AMED* Alternate Mediterranean Diet score, *APC* Adenomatous polyposis coli, *ART* Abdominal radiotherapy, *BMI* Body mass index, *cbh* Cellobiohydrolase gene, *CIMP* CpG island methylator phenotype, *CMV* Cytomegalovirus, *CRC* Colorectal cancer, *CT* Computed tomography, *DASH* Dietary Approaches to Stop Hypertension, *DM* Diabetes mellitus, *EBV* Epstein–Barr virus, *EOCRC* Early-onset colorectal cancer, *ERR* Excess relative risk, *FAP* Familial adenomatous polyposis, *FDR* First-degree relative, *HL* Hodgkin lymphoma, *HPA* Hypothalamic–pituitary–adrenal, *HPV* Human papillomavirus, *HR* Hazard ratio, *IBD* Inflammatory bowel disease, *IGF-1* Insulin-like growth factor-1, *IRS2* Insulin receptor substrate-2, *JCV* JC polyomavirus, *KEGG* Kyoto Encyclopedia of Genes and Genomes, *KO* KEGG orthology, *LINE-1* Long interspersed nuclear element-1, *LOCRC* Late-onset colorectal cancer, *MetS* Metabolic syndrome, *MMPs* Micro- and nanoplastics, *MSI* Microsatellite instability, *MR* Mendelian randomization, *MNPs* Microplastics and nanoplastics, *NSAIDs* Non-steroidal anti-inflammatory drugs, *OR* Odds ratio, *PFAS* Per- and polyfluoroalkyl substances, *PGVs* Pathogenic germline variants, *PI3K* Phosphatidylinositol-3-kinase, *pldB* Phospholipase D gene, *POPs* Persistent organic pollutants, *RR* Relative risk, *RT* Radiotherapy, *SCFAs* Short-chain fatty acids, *SO₂* Sulfur dioxide; *TGF-*β Transforming growth factor-beta, *TP53* Tumor protein p53, *UPF* Ultra-processed food, *WC* Waist circumference

## Clinical Challenges in EOCRC

EOCRC presents distinct clinical challenges compared with LOCRC, particularly with respect to symptom presentation, diagnostic delay, tumor location, and molecular characteristics, as depicted in Table 2. EOCRC is predominantly a symptom-driven disease, most commonly presenting with rectal bleeding, abdominal or pelvic pain, and changes in bowel habits. Hematochezia is reported in approximately one-third of EOCRC cases, followed by abdominal pain and altered stool frequency or caliber. In younger adults, these symptoms are often misattributed to benign conditions, such as hemorrhoids or irritable bowel syndrome, leading to delayed diagnostic evaluation [144]. Symptom-to-diagnosis intervals of approximately 7–9 months are commonly reported in EOCRC, substantially longer than in older adults. These delays reflect both patient-level factors, including reduced healthcare utilization and underinsurance, and provider-level factors, such as a lower index of suspicion and delayed referral for colonoscopic evaluation. Importantly, prolonged diagnostic delay rather than tumor aggressiveness alone appears to be a major contributor to advanced-stage presentation [4]. Consequently, EOCRC is more frequently diagnosed at advanced stages (III–IV) compared with LOCRC [4].

**Table 2:** Key differences between EOCRC and LOCRC

Features	EOCRC	LOCRC
Epidemiologic features		
Proportion of all CRC (%)	10–14% of all cases	86–90% of all cases
Incidence trend	Increasing globally (~ 2% annually)	Decreasing (1.3–4.2% annually)
Age of diagnosis	< 50 years of age	≥ 50 years of age
Family history of CRC, %	13.8–33.5%	8.3–19.3%
Clinical Presentation		
Common symptoms, %	Hematochezia (45–71%), abdominal pain (40–46%), altered bowel habits (27–54%)	Hematochezia (25–35%), abdominal pain (23–27%), often incidental diagnosis (14.6%)
Symptom-to-diagnosis interval	Longer (128–243 days)	Shorter (79–154 days)
Tumor location	Left-sided/rectal predominance (70–80%)	More evenly distributed (60% left-sided)
Pathologic features		
Stage at diagnosis	↑ advanced (stages III-IV)	↓ advanced due to routine screening
Histologic grade	↑ poorly differentiated	↓ poorly differentiated
Mucinous/signet-ring histology	↑ common	↓ common
Perineural/vascular invasion	↑ common	↓ common
Molecular Features		
MSI-H status	↑ common (10–21%), mostly Lynch syndrome	Less common (2–9%), mostly sporadic MLH1 methylation
Germline pathogenic variants	Higher (16–25%)	Lower (~ 10%)
Key mutations	More common *TP53*,* PTEN* and *CTNNB1* (especially < 40 years of age)	More common *APC*,* KRAS*,* BRAF*, and *FAM123B*
CMS	↑ CMS1 (MSI-immune subtype) particularly in < 40 years and CMS2 (canonical, WNT/MYC activation) CMS3 (metabolic) and CMS4 (mesenchymal) uncommon	-Balanced distribution - CMS2 most prevalent - CMS1 less common except in older patients (≥ 70 years) -↑ common CMS3 and CMS4 compared to EOCRC
Treatment outcomes		
Treatment intensity	Higher rates of multiagent chemotherapy, better treatment completion	Lower treatment intensity
Prognosis (stage-adjusted)	Similar to LOCRC	Similar to EOCRC
Stage IV survival	Variable (some studies show worse outcomes)	Variable
Survivorship Issues		
Fertility concerns	Major concern due to treatment effects	Less relevant
Psychosocial impact	Higher (career disruption, financial stress, family planning)	Lower
Long-term toxicity burden	Longer duration of survivorship with treatment sequelae	Shorter duration

*CRC*, colorectal cancer, *CMS* consensus molecular subtype, *EOCRC* early-onset colorectal cancer, *HR* hazard ratio, *IBD* inflammatory bowel disease, *LOCRC* late-onset colorectal cancer, *MLH1* MutL homolog 1, a DNA mismatch repair (MMR) gene;* MMR* mismatch repair, *MSI-H* microsatellite instability–high

EOCRC shows a marked left-sided predominance, with more than 70% of tumors arising in the distal colon or rectum, whereas LOCRC shows a more even distribution across the colon [4]. This anatomic pattern partly explains the predominance of rectal bleeding as a presenting symptom but also underscores the limitations of symptom-based triage in the absence of routine screening in younger adults [5].

Although EOCRC shares many somatic alterations with LOCRC, important molecular distinctions have been identified. EOCRC tumors are less likely to harbor mutations in *APC*, *KRAS*, and *BRAF*, and more likely to exhibit alterations in *TP53*, *PTEN*, and *CTNNB1*, particularly among microsatellite-stable cancers [145]. Microsatellite instability–high (MSI-H) status is more frequently observed in EOCRC than in LOCRC; however, this difference is largely attributable to a higher prevalence of Lynch syndrome in younger patients rather than sporadic MLH1 promoter hypermethylation. When Lynch syndrome–associated tumors are excluded, molecular differences between EOCRC and LOCRC become less pronounced, suggesting partial overlap in carcinogenic pathways [4]. Pathologically, EOCRC is more often associated with poor differentiation, mucinous histology, and signet-ring cell features, particularly in very young patients, as well as higher rates of lymph node involvement, lymphovascular invasion, and perineural invasion [126, 146].

Recent multi-omics studies further support the biological distinctiveness of EOCRC. At the transcriptomic level, EOCRC tumors exhibit higher expression of genes involved in Wnt/β-catenin signaling and growth factor receptor pathways, with less pronounced differential expression of DNA damage response genes compared with LOCRC [126]. Metabolomic analyses reveal that EOCRC is characterized by unique alterations in catecholamine metabolism (notably downregulation of homovanillic acid), increased phospholipid signaling, and distinct plasma metabolite profiles such as lower citrate and altered aminoadipate and uridine levels, implicating lysine biosynthesis and nucleotide metabolism pathways. These metabolic differences are not seen in LOCRC, which shows more pronounced EGFR signaling and oxidative stress-related gene changes [147, 148]. Proteomic and integrated multi-omics analyses have identified deregulated redox homeostasis through dysregulation of NRF2-mediated oxidative stress response, CXCL12-CXCR4 signaling, and glutathione metabolism as molecular hallmarks of EOCRC [21]. These omics distinctions suggest that EOCRC is biologically and molecularly unique, with implications for pathogenesis, biomarker development, and potential therapeutic targeting.

Despite more advanced stage at diagnosis, EOCRC patients often demonstrate stage-adjusted survival comparable to that of LOCRC [149]. Younger patients typically have better performance status, higher rates of treatment completion, and greater tolerance of multimodality therapy. However, these advantages do not always translate into superior outcomes, particularly in high-risk or metastatic disease, where relapse rates may be higher [150]. In addition, EOCRC patients face unique survivorship challenges, including long-term metabolic, cardiovascular, reproductive, and psychosocial consequences of treatment. Fertility impairment, chronic bowel dysfunction, sexual dysfunction, and body image distress are common and represent important quality-of-life considerations in this younger population [21].

Collectively, these clinical and molecular features define EOCRC as a diagnostically vulnerable yet biologically distinct entity, underscoring the need for increased symptom awareness, earlier diagnostic evaluation, and integration of molecular and metabolic markers into individualized risk assessment and management.

## Preventive Strategies and Future Directions

**Fig. 3:**
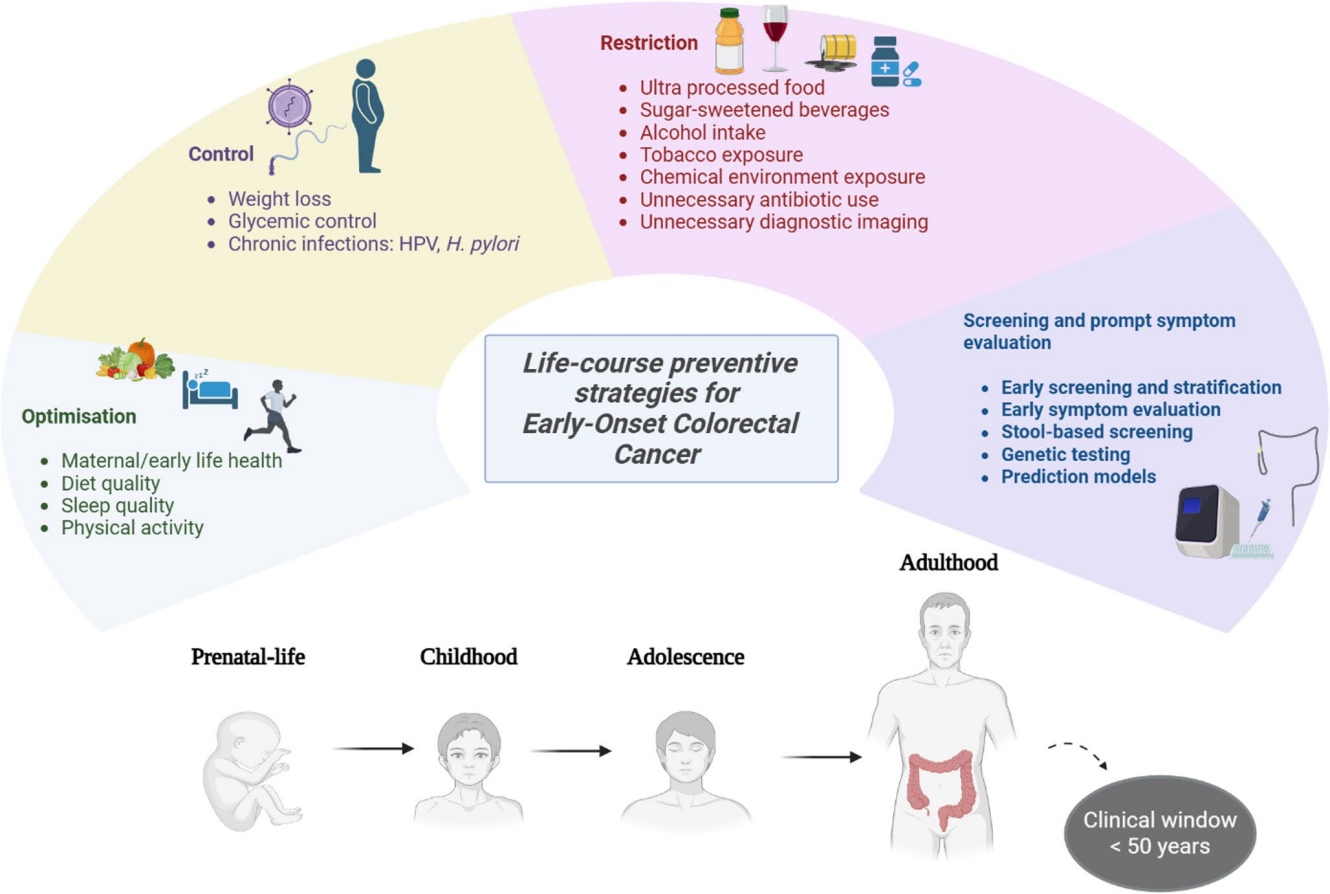
Life-course preventive strategies for EOCRC. Created by Dimitra Petropoulou in BioRender (January 20, 2026) https://BioRender.com/h7nxebv

The rising incidence of EOCRC necessitates a public health response that extends beyond traditional age-based screening paradigms. Given the multifactorial and life-course nature of EOCRC, effective prevention requires integrated strategies spanning early detection, lifestyle modification, medical intervention, and targeted research, as depicted in Figure 3.

### Screening, risk prediction and prompt symptom evaluation

Lowering the recommended age for average-risk CRC screening to 45 years represents an important step forward; however, this strategy alone is insufficient to address EOCRC risk, as a substantial proportion of cases occur before routine screening eligibility [151]. Colonoscopy remains the gold standard for CRC detection and prevention through adenoma removal, particularly given the distal predominance of EOCRC. Exclusive reliance on colonoscopy may exacerbate disparities in access, adherence, and healthcare utilization [152].

Noninvasive stool-based tests, including fecal immunochemical testing (FIT) and multitarget stool DNA assays, offer scalable alternatives for population-level screening and may serve as interim or adjunctive tools for younger adults at increased risk. Although these tests are validated primarily in individuals aged ≥ 45 years, expanding their use in risk-stratified populations younger than 50 years represents a pragmatic approach to earlier detection [153]. There is also increasing interest in multivariable risk prediction models that incorporate family history, metabolic factors (obesity, diabetes), lifestyle exposures (diet, alcohol, smoking), and emerging biomarkers. Such models could enable risk-adapted screening, prioritizing colonoscopy or intensive surveillance for individuals at highest risk before age-based thresholds [152].

Universal tumor testing for mismatch repair deficiency has improved the identification of Lynch syndrome, yet germline testing remains underutilized in younger patients. Broader implementation of germline multigene panel testing in EOCRC could refine risk stratification for both patients and relatives [154]. Emerging biomarkers, including circulating tumor DNA, methylation-based blood assays, and microbiome-derived signatures, hold promise for early detection, although their role in population screening remains investigational. Importantly, such tools may eventually enable non-invasive surveillance in high-risk young adults unwilling or unable to undergo colonoscopy [155].

Overall, EOCRC remains largely symptom-driven. Rectal bleeding, abdominal pain, and changes in bowel habits are frequently dismissed in younger adults, leading to diagnostic delay. Public health and clinical efforts should therefore emphasize symptom awareness and timely evaluation, particularly in primary care settings and underserved populations [4].

### Lifestyle and Environmental Interventions

Dietary patterns represent one of the most modifiable EOCRC risk factors. Multiple cohort studies and systematic reviews consistently demonstrate that Western dietary patterns, characterized by high intake of processed meats, refined carbohydrates, SSBs, and UPFs, are associated with increased risk of EOCRC and its precursor lesions, particularly high-risk distal and rectal adenomas [18, 156]. Conversely, higher adherence to Mediterranean, Dietary Approaches to Stop Hypertension (DASH), and Alternative Healthy Eating dietary patterns has been associated with reduced risk of early-onset colorectal adenomas, precursors of EOCRC (Table 1) [45].

Public health strategies should promote whole-food, fiber-rich dietary patterns while discouraging excessive intake of UPFs and SSBs. Policy-level interventions, such as front-of-package labeling, taxation of SSBs, and food reformulation incentives, may achieve broader population impact than individual counseling alone [157].

Microbiome-targeted approaches, including probiotics, postbiotics, and time-restricted eating, may show promise for restoring eubiosis, reducing intestinal inflammation, and improving metabolic and immune profiles relevant to EOCRC pathogenesis [65, 158, 159]. Protective dietary components, including dietary fiber, calcium, folate, β-carotene, and vitamins C, D and E, as well as foods such as yogurt and coffee, have been associated with reduced CRC risk and may counteract metabolic and inflammatory pathways relevant to EOCRC [18, 160]. Precision nutrition, tailored to individual risk profiles and molecular features, represents a promising strategy for EOCRC prevention and management, but further research is required to define best practices and clinical impact. Moreover, sustained weight loss, especially when achieved early in adulthood, is expected to reduce the risk of EOCRC, although prospective data specific to EOCRC remain limited [18]. However, unintentional weight loss preceding diagnosis may be a clinical sign of EOCRC rather than a protective factor [161].

Population-level reduction of alcohol consumption and smoking remains essential. Increasing physical activity and reducing sedentary time represent additional intervention targets. Digital health tools and workplace interventions may support behavior change at scale. Improving sleep quality and circadian health, including reduction of night-time light exposure, represents an underrecognized but potentially relevant prevention strategy [18].

Reducing exposure to environmental and chemical risk factors, including MNPs, persistent organic pollutants, pesticides, and unnecessary medical radiation, represents an emerging prevention frontier. While individual effect sizes are modest, the ubiquity and early-life onset of these exposures support precautionary public health approaches, including improved food packaging regulation, safer materials, and judicious use of diagnostic imaging, particularly in children and young adults [162]. Antibiotic stewardship, especially in pediatric and adolescent populations, may yield long-term cancer-preventive benefits beyond antimicrobial resistance, given the role of early-life microbiome disruption in EOCRC susceptibility [163].

Given mounting evidence for early-life epigenetic programming, attention to maternal health, nutrition, and metabolic status, along with infant feeding practices (e.g. plastic bottles) and early antibiotic exposure, may influence EOCRC risk decades later. These considerations reinforce the need for a life-course approach to prevention [164].

### Surgical and Pharmacologic Interventions

Bariatric surgery has been associated with reduced CRC risk in individuals with severe obesity, likely through durable improvements in insulin sensitivity and inflammation, with meta-analyses reporting RR reductions of ~ 35–50% compared with nonsurgical management [165]. However, EOCRC-specific data remain limited, and recent analyses have not shown a clear reduction in EOCRC incidence following bariatric surgery [166].

Several commonly used medications may modify EOCRC risk through metabolic or anti-inflammatory pathways. GLP-1 receptor agonists improve weight control and insulin sensitivity, and may have downstream cancer-preventive effects, though long-term EOCRC data are not yet available. Metformin has been associated with reduced colorectal neoplasia risk in diabetic populations, while statins, aspirin, and NSAIDs show chemopreventive effects in CRC overall [167–169]. However, EOCRC-specific evidence is insufficient to support population-level recommendations, and careful risk–benefit assessment is required. Eradication of *H. pylori* reduces gastric cancer risk and may influence colorectal carcinogenesis through systemic inflammatory pathways. Although EOCRC-specific evidence is limited, infection control remains a reasonable adjunct to broader cancer and CRC prevention strategies [123].

### Future Research Priorities

A critical gap in EOCRC prevention is the lack of large, prospective, age-specific cohorts with detailed exposure assessment. Future studies must capture early-life, metabolic, dietary, environmental, infectious and microbiome data to disentangle timing and causality. Integration of genomics, epigenomics, transcriptomics, metabolomics, and microbiomics with longitudinal exposure histories may identify early biomarkers of risk and mechanistic targets for intervention. Most EOCRC research has been conducted in high-income, Western populations. Expanding studies to diverse global populations is essential to understand how rapid lifestyle transitions influence EOCRC risk and to avoid widening health disparities.

### HPV: A Research Priority Beyond Metabolic Research

HPV represents an underexplored exposure in EOCRC. Although evidence linking HPV to colorectal carcinogenesis is heterogeneous and causality remains unproven due to study limitations and confounding, its established oncogenic role in other anogenital cancers and the rising incidence of EOCRC in younger adults warrant targeted investigation. Future studies should integrate tumor HPV testing (PCR, genotyping, and oncoprotein detection), vaccination status, sexual health data, and long-term outcomes to clarify causality, ideally using prospective cohorts with incident-tumor biobanking [170]. Non-invasive HPV diagnostics (e.g. urine and serum exosome HPV testing) may facilitate large-scale screening and mechanistic studies [171]. If HPV relevance to EOCRC is confirmed, widespread HPV vaccination, particularly the 9-valent vaccine covering ~ 90% of HPV-attributable cancers, could be leveraged as a population-level prevention strategy, especially with expanded vaccination across sexes and age groups [172–174]. HPV vaccines are highly effective in preventing HPV-related malignancies, and emerging cohort data suggest possible reductions in non-cervical cancers; however, no significant protection against rectal cancer has yet been demonstrated, likely reflecting long latency periods [174].

Overall, effective EOCRC prevention requires a shift from age-based screening alone toward integrated, life-course–oriented strategies. By combining risk-adapted early detection with lifestyle, environmental, and metabolic interventions, public health efforts may move from reactive diagnosis to proactive prevention.

## Limitations, Challenges and Controversies

Current research on EOCRC etiology is constrained by several critical methodological limitations. Most evidence derives from high-income countries, particularly North America and Europe, limiting the generalizability of findings to diverse global populations experiencing rising EOCRC incidence [3]. Meta-analyses examining EOCRC risk factors show substantial heterogeneity (I² >60%), reflecting inconsistencies in study design, exposure measurement, and population characteristics [18]. A central limitation is the predominance of retrospective and case–control designs, which are vulnerable to recall bias (diet, antibiotics, stress), reverse causation (prodromal symptoms influencing behavior), and selection bias. EOCRC definitions vary across studies, with age cutoffs ranging from < 40 to < 55 years, and outcomes differ across analyses (adenomas vs. cancer; colon vs. rectum) complicating cross-study comparability. Exposure assessment methods are heterogeneous and often rely on self-report or ecological proxies rather than objective biomarkers, increasing misclassification risk. The relatively low absolute number of EOCRC cases compared with late-onset disease further limits statistical power for subgroup analyses and gene–environment interaction studies, resulting in imprecise estimates and unstable effect sizes [76]. Consequently, observed effect sizes are typically modest, fueling debate regarding clinical translation and prioritization of preventive interventions. Moreover, while metabolic dysfunction, obesity, diabetes, alcohol consumption, smoking, and sedentary behavior show relatively consistent EOCRC-specific associations, several environmental and infectious exposures discussed in this review remain exploratory and should be interpreted with caution.

Despite identification of multiple risk factors, fundamental gaps remain in understanding EOCRC causality. In particular, several exposures discussed in this review (e.g. microplastics, viral detection studies) report large effect sizes in small or cross-sectional studies; these findings require cautious interpretation until validated in prospective, exposure-based cohorts. An additional methodological concern relates specifically to the analytical detection and quantification of MNPs in human biospecimens. Recent analytical reviews highlight substantial challenges in reliably identifying and characterizing MNPs in complex biological matrices, including risks of sample contamination, limitations in sensitivity and specificity of vibrational spectroscopy techniques, interference from endogenous biological compounds, and lack of standardized reference materials and harmonized reporting criteria [175]. Many currently applied techniques provide incomplete morphological or chemical characterization, and orthogonal confirmation using complementary analytical approaches is not uniformly implemented. As a result, uncertainty remains regarding exposure quantification, particle size distribution, and polymer specificity, which may influence risk estimation and interpretation of reported associations. These analytical limitations underscore the need for standardized protocols, contamination control procedures, and transparent reporting frameworks when evaluating MNP-related carcinogenic risk [175].

Beyond analytical challenges, prospective cohorts beginning in early life, adolescence, or pregnancy are limited, and repeated, high-quality exposure measurements across the life course are rarely available. Exposures hypothesized to be critical, such as early-life antibiotic use, childhood obesity trajectories, pubertal metabolic changes, and cumulative dietary patterns, are poorly captured in adult cohorts. As a result, temporal relationships between early exposures and EOCRC development later remain inadequately characterized. The lack of longitudinal studies integrating early-life exposures with biospecimen collection and multi-omics profiling limits identification of critical windows of susceptibility [176, 177].

Several controversies persist regarding the relative importance and mechanisms of proposed EOCRC risk factors. Obesity shows sex- and site-specific heterogeneity with stronger associations observed in men and for colon versus rectal cancers [29]. Moreover, rising EOCRC incidence in East Asian countries with relatively low obesity prevalence suggests that factors beyond body mass, such as dietary composition, UPF consumption, and other environmental exposures, may play a more prominent role [3]. Similarly, while gut dysbiosis is consistently observed in EOCRC, it remains unclear whether microbial alterations are causal drivers, early facilitators, or downstream consequences of carcinogenesis. Another recurring controversy is whether EOCRC represents a single entity or a heterogeneous mixture of diseases unified only by age at diagnosis. The distribution of tumor location (distal/rectal predominance), mismatch repair status, and molecular subtypes varies across cohorts, raising the possibility that some risk factors are site-specific (rectal vs. proximal colon) or subtype-specific (chromosomal instability vs. MSI-high tumors).

Another major challenge is the limited investigation of gene–environment and epigenetic interactions. Although genetic susceptibility and environmental exposures have been independently associated with EOCRC, their interaction remains poorly investigated. Epigenetic acceleration is a promising integrative approach, yet systematic studies of environmentally induced epigenetic changes across tissues and developmental stages are still limited.

Finally, EOCRC research faces challenges in quantifying complex exposome factors. UPFs, endocrine-disrupting chemicals, MNPs, radiation, and air pollution are often assessed using proxies rather than individual-level exposure data, increasing uncertainty. Antibiotic studies are confounded by indication (infections and inflammation), and microbiome studies may be influenced by reverse causation or treatment effects. Despite these limitations, convergence of evidence across epidemiologic, mechanistic, and multi-omics studies supports the biological plausibility of a multifactorial EOCRC model.

## Conclusion

The rising incidence of EOCRC represents a shift in CRC epidemiology, and this review proposes an integrated life-course framework that consolidates metabolic, exposomic, microbial, epigenetic, and societal determinants into a unified etiologic model. Accumulating evidence indicates that EOCRC cannot be adequately explained by hereditary syndromes or traditional risk factors alone. Instead, EOCRC emerges as a multifactorial, life-course disease shaped by cumulative interactions between genetic susceptibility and early, persistent metabolic, environmental, microbial, and societal exposures. This model reconciles strong birth-cohort effects with long premalignant latency and helps explain why EOCRC incidence continues to rise despite stable or declining rates in older populations.

A central unifying theme of this review is metabolic and inflammatory stress as a key biological substrate linking modern exposures to premature colorectal carcinogenesis. Obesity, insulin resistance, UPFs, sedentary behavior, alcohol consumption, and gut microbiome dysbiosis converge on shared pathways, including insulin/IGF signaling, chronic low-grade inflammation, epithelial barrier dysfunction, immune dysregulation, and genotoxic stress, that lower the threshold for malignant transformation at younger ages. These processes interact with epigenetic mechanisms, leading to accelerated epigenetic aging that precedes clinical disease by decades.

Beyond metabolic factors, this review highlights the relevance of the modern exposome, encompassing environmental pollutants, MNPs, persistent organic chemicals, radiation, antibiotics, and chronic infections. Although individual effect sizes are generally modest, their ubiquity, early-life onset, and cumulative nature may confer substantial population-level risk. Many of these exposures remain underrecognized in clinical risk assessment and prevention strategies.

Clinically, EOCRC is characterized by symptom-driven diagnosis, delayed evaluation, and distal tumor predominance, contributing to advanced-stage presentation despite comparable stage-adjusted survival to later-onset disease. These features underscore that delayed detection, rather than intrinsic treatment resistance, remains a major contributor to EOCRC burden. These observations reinforce the need for risk-adapted screening, prompt symptom evaluation, and improved access to care, particularly for younger adults and underserved populations.

Looking forward, meaningful progress in EOCRC prevention will require a shift from age-based paradigms toward integrated, risk-adapted, life-course approaches. Priorities include the development of prospective EOCRC-specific cohorts, incorporation of multi-omics and exposomic data, validation of noninvasive biomarkers, and rigorous investigation of gene–environment and epigenetic interactions. Translation of current knowledge into public health action, through improved diet quality, antibiotic stewardship, reduction of avoidable radiation exposure, promotion of physical activity and sleep health, and earlier diagnostic evaluation, will be essential. In summary, EOCRC should be viewed as an early manifestation of cumulative metabolic and environmental stress acting on genetically susceptible individuals. Addressing its rising incidence demands an integrated strategy bridging molecular epidemiology, clinical medicine, and public health, with a focus on proactive prevention rather than reactive treatment across the lifespan. 

## Key references

- Hua H, Jiang Q, Sun P, Xu X. Risk factors for early-onset colorectal cancer: systematic review and meta-analysis. Front Oncol. 2023;13:1132306. 10.3389/fonc.2023.1132306. (of outstanding importance).

- This meta-analysis provides an overview of the main risk factors of EOCRC.

- Khoa Ta HD, Nguyen NN, Ho DKN, Nguyen HD, et al. Association of diabetes mellitus with early-onset colorectal cancer: A systematic review and meta-analysis of 19 studies including 10 million individuals and 30,000 events. Diabetes Metab Syndr. 2023;17(8):102828.10.1016/j.dsx.2023.102828 . (of importance).

- This meta-analysis provides evidence that diabetes mellitus is a risk factor for EoCRC

- Zheng X, Hur J, Nguyen LH, Liu J, Song M, Wu K, et al. Comprehensive Assessment of Diet Quality and Risk of Precursors of Early-Onset Colorectal Cancer. J Natl Cancer Inst. 2021 May 4;113(5):543-552. 10.1093/jnci/djaa164. (of importance).

- In this prospective cohort study (Nurses' Health Study II), it was shown that poor diet quality was linked to an elevated risk of early-onset distal and rectal adenomas of high malignant potential.

- Deng JW, Zhou YL, Zhang YX, Zhou CB, Fang JY. The relationship between gut microbiota, lifestyle habits, and early-onset colorectal cancer: shedding light on early prediction. Br J Cancer. 2025 (in press). 10.1038/s41416-025-03277-x .(of importance).

- In this observational study, it was found that EO-CRC-enriched bacteria could mediate the effects of lifestyle and dietary factors on colorectal carcinogenesis.

- Laskar RS, Murphy N, Ferrari P, Brennan P, Cross AJ, Guevara M, Pala V, et al. A prospective investigation of early-onset colorectal cancer risk factors-pooled analysis of three large-scale European cohorts. Br J Cancer. 2025 (in press). 10.1038/s41416-025-03303-y. (of importance).

## Supplementary Information

Below is the link to the electronic supplementary material.

Supplementary Material 1 (PDF 322 KB)

Supplementary Material 2 (PDF 244 KB)

## Data Availability

No datasets were generated or analysed during the current study.

## Abbreviations

APC: adenomatous polyposis coli
BMI: body mass index
Cbh: cellobiohydrolase gene
CIMP: CpG island methylator phenotype
CMS: consensus molecular subtype
CRC: colorectal cancer
ctDNA: circulating tumor DNA
EOCRC: early-onset colorectal cancer
ERR: excess relative risk
FAP: familial adenomatous polyposis
FDR: first-degree relative
FIT: fecal immunochemical test
GI: gastrointestinal
GLP-1: glucagon-like peptide-1
GWAS: genome-wide association study
HPV: human papillomavirus
HR: hazard ratio
HPA: hypothalamic–pituitary–adrenal
IBD: inflammatory bowel disease
IGF-1: insulin-like growth factor-1
KEGG: Kyoto Encyclopedia of Genes and Genomes
KO: KEGG orthology
LINE-1: long interspersed nuclear element-1
LOCRC: late-onset colorectal cancer; MetS, metabolic syndrome
MLH1: MutL homolog 1
MMR: mismatch repair
MNPs: micro- and nanoplastics
MSI-H: microsatellite instability–high
NGS: next-generation sequencing
NSAIDs: non-steroidal anti-inflammatory drugs
OR: odds ratio
PFAS: per- and polyfluoroalkyl substances
PGVs: pathogenic germline variants
PI3K: phosphatidylinositol-3-kinase
pldB: phospholipase D gene
POPs: persistent organic pollutants
RR: relative risk
RT: radiotherapy
SCFAs: short-chain fatty acids
T2DM: type 2 diabetes mellitus
TGF-β: transforming growth factor-β
TNM: Tumor–Node–Metastasis
TP53: tumor protein p53
UPFs: ultra-processed foods
